# Supplementary material for: Eco-Friendly Water-Processable Polyimide Binders with High Adhesion to Silicon Anodes for Lithium-Ion Batteries
Source: Nanomaterials (Basel). 2021 Nov 23;11(12):3164. doi: 10.3390/nano11123164 (PMC8705944; doi:10.3390/nano11123164)
Supplement: Supplementary file 1 [file nanomaterials-11-03164-s001.zip › nanomaterials-1447812-supplementary.pdf]

Supporting Information

Eco-Friendly Water-Processable Polyimide Binders with High Adhesion to Silicon Anodes for Lithium-Ion Batteries

Yujin So [1,2,†], Hyeon-Su Bae [3,†], Yi Young Kang [1], Ji Yun Chung [1,4], No Kyun Park [1], Jinsoo Kim [1], Hee-Tae Jung [2], Jong Chan Won [1,4,*], Myung-Hyun Ryou [3,*] and Yun Ho Kim [1,4,*]

[1] Advanced Materials Division, Korea Research Institute of Chemical Technology (KRICT), Daejeon 34114, Korea; soyujin@krict.re.kr (Y.S.); yykang@krict.re.kr (Y.Y.K.); jjyjjy@krict.re.kr (J.Y.C.); nkpark@krict.re.kr (N.K.P.); jinsoo@krict.re.kr (J.K.)

[2] Department of Chemical and Biomolecular Engineering, Korea Advanced Institute of Science and Technology (KAIST), Daejeon 34141, Korea; heetae@kaist.ac.kr

[3] Department of Chemical and Biological Engineering, Hanbat National University, Daejeon 34158, Korea; hsbae1234@gmail.com

[4] Korea Research Institute of Chemical Technology (KRICT) School, University of Science and Technology, Daejeon 34113, Korea

* Correspondence: jcwon@krict.re.kr (J.C.W.); mhryou@hanbat.ac.kr (M.-H.R.); yunho@krict.re.kr (Y.H.K.); Tel.: +82-860-7294 (J.C.W.); +82-42-821-1534 (M.-H.R.); +82-42-860-7274 (Y.H.K.)

† These authors contributed equally to this work.

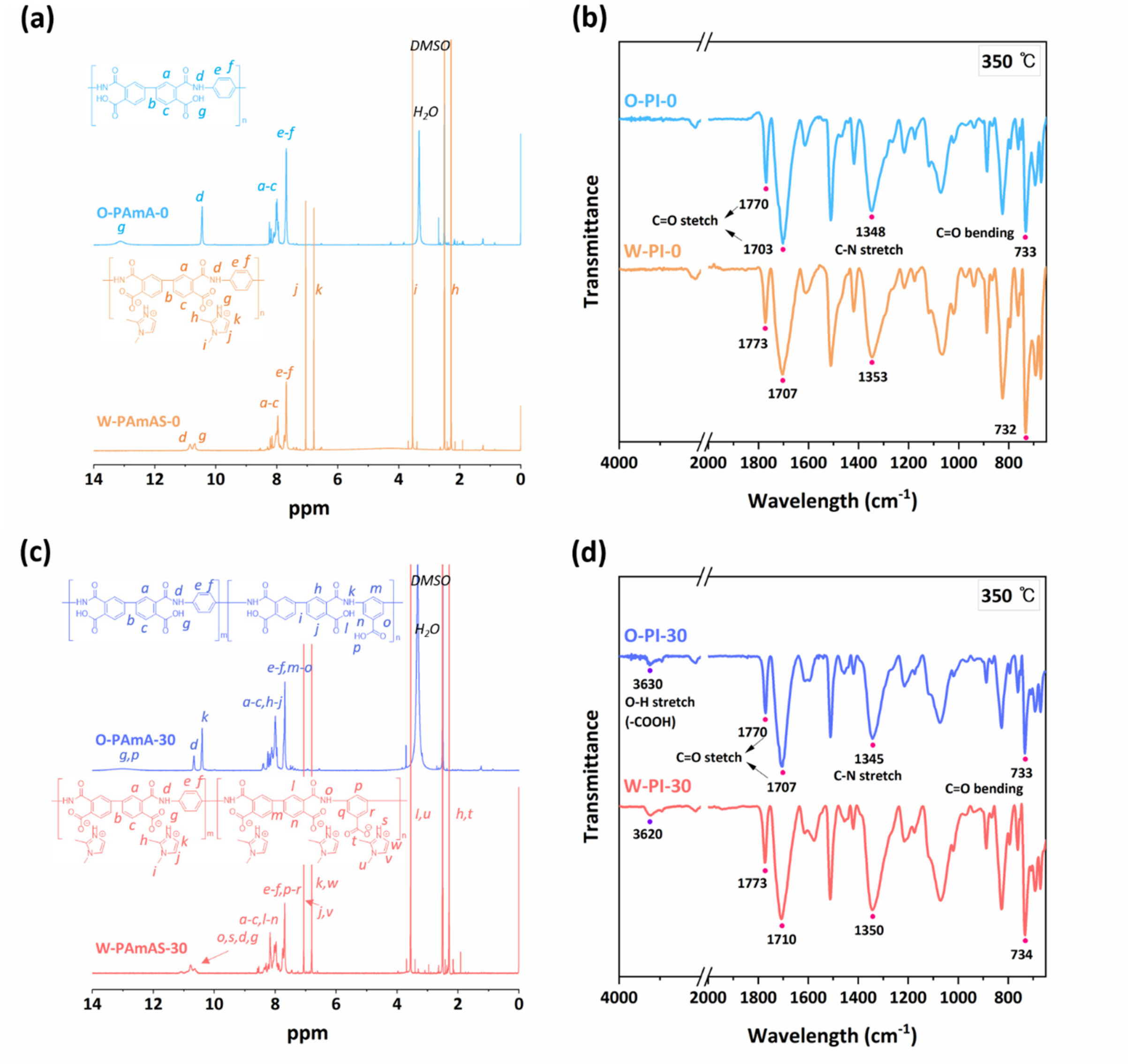

Figure S1. ^{1}H NMR spectra of (**a**) O-PAmA-0 and W-PAmA-0 and (**c**) O-PAmA-30 and W-PAmA-30 in DMSO-d6. FT-IR spectra (**b**) O-PI-0 and W-PI-0 and (**d**) O-PI-30 and W-PI-30 after imidization at 350 °C.

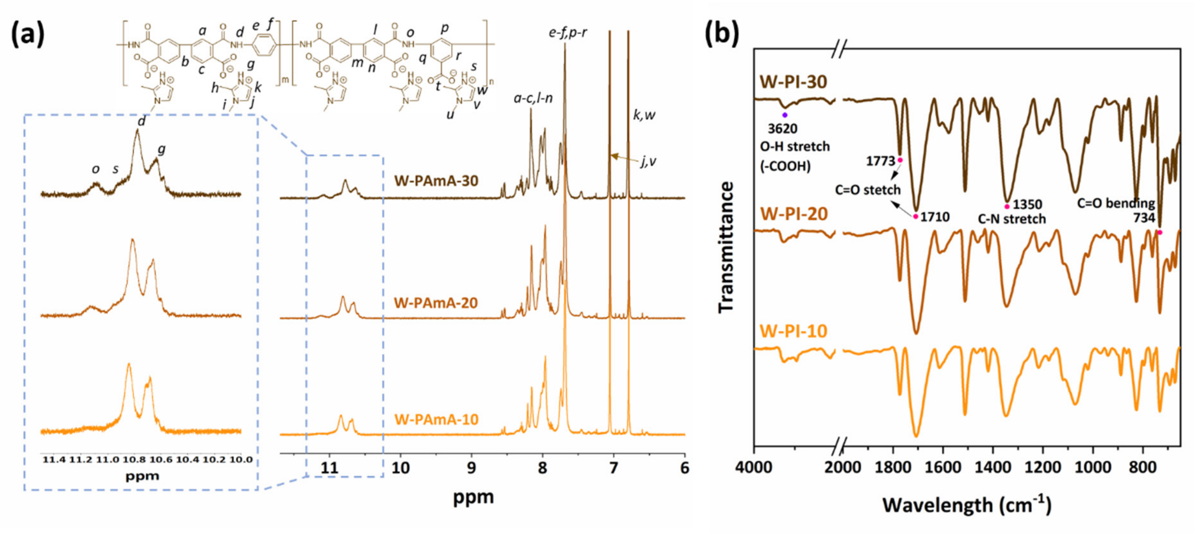

Figure S2. (**a**) ^{1}H NMR spectra of W-PAmAS-# and (**b**) FT-IR spectra of W-PI-# in proportion to the DABA content.

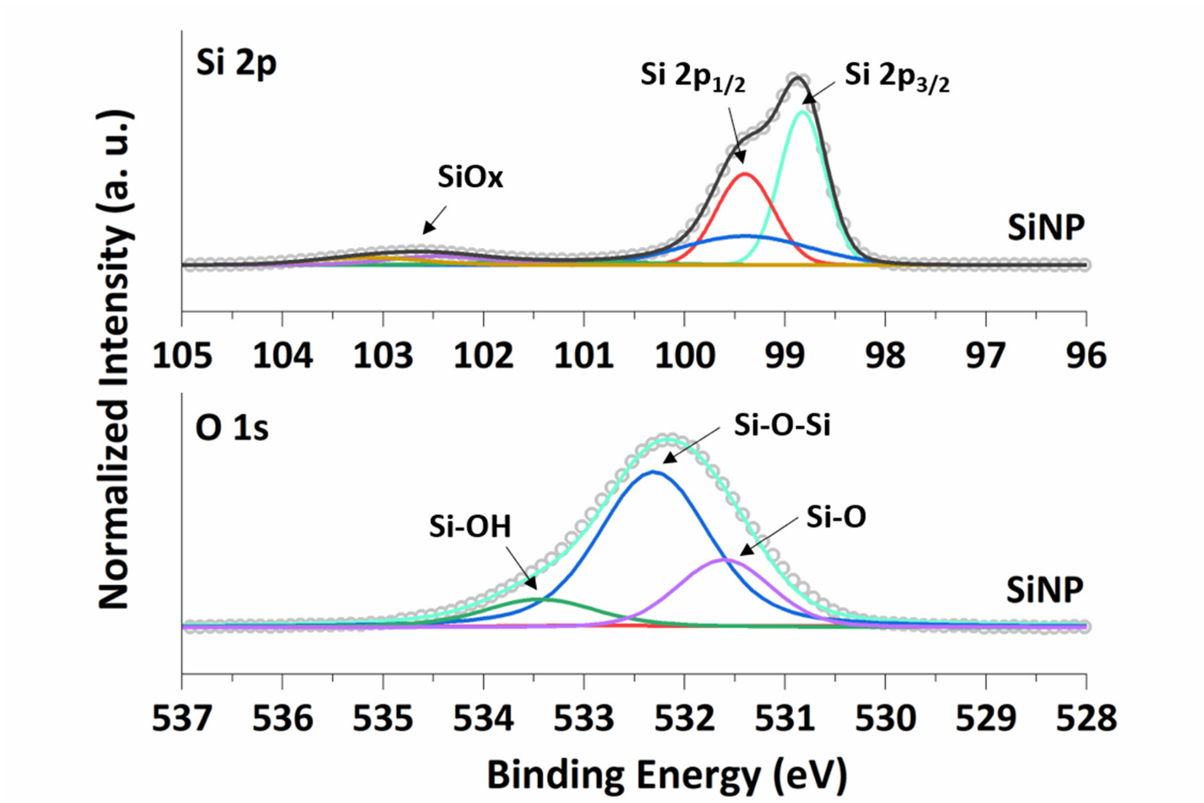

Figure S3. XPS spectra of Si 2p and O 1s in SiNPs.

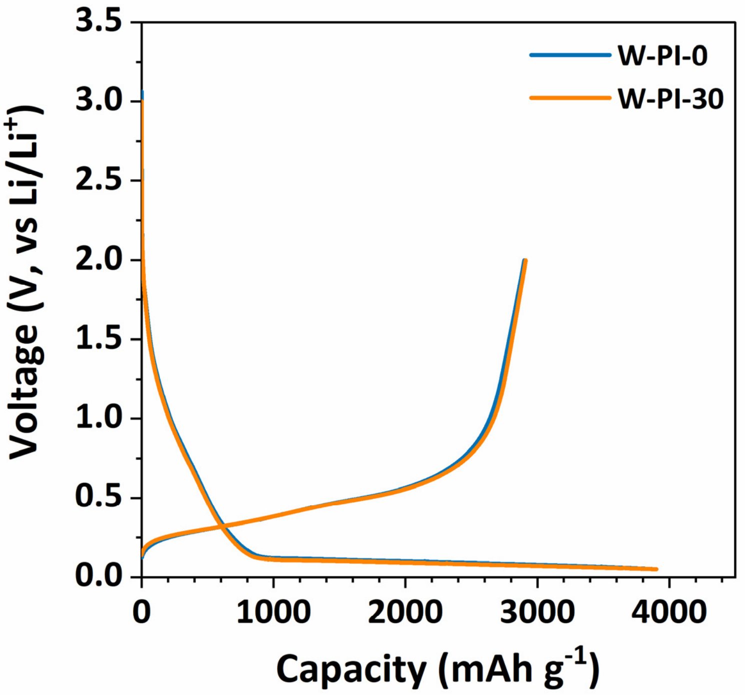

Figure S4. Precycling curves of Si anodes with W-PI binders (0.005–2.0 V vs. Li/Li^+, 200 mA g^{-1} at 25 °C).

Table S1. Cycle performance of Si-based anodes with different polymer binders

Binder	Anode material	Specific Capacity (mAh g^{-1})	Retention (%)	Current rate	Content of binder (%)	Ref.
Our polyimide (W-PI-30)	***Nano-Si***	***2061***	***1883 mAh g^{-1} (91.3 %) (200 cycles)***	***1200 mA g^{-1}***	***20***	**-**
**Carboxymethyl chitosan**	Si	1990	950 mAh g^{-1} (50 cycles)	500 mA g^{-1}	8	[22]
**PVA-PEI**	Nano-Si	3072.9	1063 mAh g^{-1} (300 cycles)	1 A g^{-1}	20	[23]
**PAA-PANI**	Nano-Si	1979	56.5 % (300 cycles)	1.0 C	25	[24]
**PPyMAA**	Nano-Si	3928.8	2200 mAh g^{-1} (over 180 cycles)	420 mA g^{-1} (0.1 C)	10	[25]
**Gum arabic (GA)**	Nano-Si	4056	2708 mAh g^{-1} (75.7%) (100 cycles)	420 mA g^{-1} (0.1 C)	25	[26]
**Polyimide**	Si	~800 (after 2nd cycle)	800 mAh g^{-1} (195 cycles)	800 mA g^{-1}	15	[41]
**Polyimide**	Si	2077	75.9 % (20 cycles)	200 mA g^{-1}	10	[42]
**Polyimide**	Si	1195.6	93.6 % (30 cycles)	0.1 C	25	[43]
**Copolyimide (P84)**	Si	1929	1313 mAh g^{-1} (300 cycles)	1.2 A g^{-1}	20	[4]
**Copolyimide (PI-200)**	Si	2989.7	2235.5 mAh g^{-1} (200 cycles)	420 mA g^{-1} (0.1 C)	20	[44]
**Polyamide imide (PAI)**	Nano-Si	~2000	1700 mAh g^{-1} (20 cycles)	0.56 mA cm^{-2}	10	[45]
